# Adaptive List Flip Decoder for Polar Codes with High-Order Error Correction Capability and a Simplified Flip Metric

**DOI:** 10.3390/e24121806

**Published:** 2022-12-10

**Authors:** Yansong Lv, Hang Yin, Zhanxin Yang, Yuhuan Wang, Jingxin Dai

**Affiliations:** 1 The Engineering Research Center of the Digital Audio and Video Ministry of Education, Communication University of China, Beijing 100024, China; 2 The State Key Laboratory of Media, Convergence and Communication, Communication University of China, Beijing 100024, China

**Keywords:** polar code, list flip decoder, flip metric, adaptive list, high-order error correction capability

## Abstract

Designing an efficient decoder is an effective way to improve the performance of polar codes with limited code length. List flip decoders have received attention due to their good performance trade-off between list decoders and flip decoders. In particular, the newly proposed dynamic successive cancellation list flip (D-SCLF) decoder employs a new flip metric to effectively correct high-order errors and thus enhances the performance potential of present list flip decoders. However, this flip metric introduces extra exponential and logarithmic operations, and the number of these operations rises exponentially with the increase in the order of error correction and the number of information bits, which then limits its application value. Therefore, we designed an adaptive list flip (ALF) decoder with a new heuristic simplified flip metric, which replaces these extra nonlinear operations in the original flip metric with linear operations. Simulation results show that the simplified flip metric does not reduce the performance of the D-SCLF decoder. Moreover, based on the in-depth theoretical analyses of the combination of the adaptive list and the list flip decoders, the ALF decoder adopts the adaptive list to further reduce the average complexity.

## 1. Introduction

Polar codes [1] have been widely studied since they were proposed due to their excellent performance. Consequently, polar codes have been selected as the control channel coding scheme in the 5G enhanced Mobile Broadband (eMBB) scenario [2] and are expected to still be competitive coding technologies in the future. In particular, polar codes using a successive cancellation (SC) decoder comprise the first channel coding technique that has been proven to achieve channel capacity with an infinite code length [1]. However, the error-correction performance of polar codes using the SC decoder with limited code length is unsatisfactory. Therefore, improving the performance of polar codes with limited code length has always been a concern of scholars, and designing an efficient decoder is one of the most effective ways to solve this problem.

The SC list (SCL) [3,4] and CRC-aided SCL (CA-SCL) [5,6] are two improved list decoders based on the SC decoder. Compared with the single candidate path of the SC decoder, they adopt a list of multiple candidate paths and posterior probability to improve the error-correction performance. In particular, the CA-SCL decoder can utilize CRC to filter wrong candidate paths on the list and obtain better performance than the SCL decoder. However, this list containing multiple candidate paths requires more computational complexity (energy) and storage space. To further optimize the CA-SCL decoder, many scholars have proposed different improved list decoders. The authors of [7] proposed a new path metric that is beneficial to hardware implementation for the filtering of candidate paths and is widely used nowadays. Ref. [8] combined the adaptive list and CRC to further reduce the average complexity of the CA-SCL decoder without loss of error-correction performance. In [9,10], the authors designed different distributed CRCs for the early termination of the CA-SCL decoder. The authors of [11] designed a threshold to reduce redundant calculations during list decoding. Furthermore, Ref. [12] proposed a polar decoder supported by parity check (PC), which has an error-correction performance comparable to that of the CA-SCL decoder. Although the list decoders above improve the performance of the CA-SCL decoder to a certain extent, their error-correction performance is similar to or even lower than that of the CA-SCL decoder with the same list size.

In addition to list decoders, Ref. [13] proposed the first flip decoder called SC flip (SCFlip). The core idea of this flip decoder is to use additional SC decoding attempts to perform flip operations in the error-prone positions, and thus the flip decoder can achieve better error-correction performance than the SC decoder. Subsequently, many scholars have contributed to improving the performance of the flip decoder. Ref. [14] effectively obtained the error-prone positions to enhance the efficiency of flip operations. Ref. [15] designed the DSCFlip decoder, which employsa new flip metric to correct high-order errors and further improve the error-correction performance of flip decoders. Ref. [16] simplified the flip metric of the DSCFlip decoder to improve its application value. Moreover, Ref. [17] first introduced parity-check bits into flip decoders to obtain a more efficient early termination capability. Their advantages are that they can achieve the performance of the CA-SCL decoder by using only a single path, and their average computational complexity is close to that of the SC decoder at a high signal-to-noise ratio (SNR). However, the computational complexity of these flip decoders may be higher than that of the CA-SCL decoder with the same error-correction performance at a low SNR.

Reference [18] proposed the first list flip decoder called the SCL bit-flip (SCL-BF) decoder. This decoder creates a bit-flip operation in list decoding processes, which combines the flip idea in flip decoders and the list in list decoders; it then realizes a good performance trade-off between the list decoders and the flip decoders. For brevity, we use the term “list flip operation” to refer to the flip operation of a list flip decoder. On the one hand, the SCL-BF decoder can obtain the error-correction performance of the CA-SCL decoder with a large list by utilizing a smaller list. On the other hand, the SCL-BF decoder can use a list of multiple candidate paths to increase the probability of keeping the correct path. However, the list flip operation and the selection of error-prone positions in the SCL-BF decoder are very similar to those in the SCFlip decoder, which means that it is not efficient enough for correcting errors in a more complex list decoding scheme. To further improve the performance gain brought by list flip operations, [19,20] proposed different flip metrics to optimize the original selection of error-prone positions, and [21] proposed a list decoder with shift-pruning (SCL-SP) to optimize the original list flip operations in the SCL-BF decoder. Moreover, Refs. [22,23] proposed different list flip decoders to optimize the shifted-pruning operation in the SCL-SP decoder. However, these list flip decoders cannot effectively flip high-order errors, which limits the error-correction performance potential of list flip decoders. A high-order error means that correcting this error requires multiple flip operations in one decoding attempt.

Recently, Ref. [24] proposed a dynamic SCL-Flip (D-SCLF) decoder with a high-order error correction capability. This decoder employsa new flip metric to estimate the likelihood of high-order errors in list decoding processes; it can therefore correct high-order errors in these list decoding processes. However, compared to the flip metric in [20], this new flip metric includes additional exponential and logarithmic operations. Furthermore, the number of these exponential and logarithmic operations grows exponentially with the order of error correction (the maximum number of list flip operations in one decoding attempt) and the number of information bits, which then reduces the application value of the list flip decoder with high-order error correction capacity.

To further develop the list flip decoder for polar codes, we designed an adaptive list flip (ALF) decoder with high-order error correction capability and a simplified flip metric. The contributions of this decoder are as follows:

- We designed a new simplified flip metric. This simplified flip metric replaces the additional exponential and logarithmic operations introduced by the original flip metric with multiplication and addition operations. The simulation results show that the D-SCLF decoder using the simplified metric has similar error-correction and complexity performance to the D-SCLF decoder using the original flip metric.
- We theoretically analyzed the characteristics of the combination of the adaptive list and list flip decoders, and we verified these theoretical analyses by simulation.
- We proposed an ALF decoder with a high-order error correction capability. The decoder mainly combines the simplified flip metrics and the adaptive list. Simulation results show that our ALF decoder can effectively reduce the average computational complexity (energy) of the D-SCLF decoder without diminishing the error-correction performance.

The remainder of the paper is organized as follows. Section 2 briefly gives an overview of the polar encoding method and some polar decoding algorithms. Section 3 describes and analyzes the details of the proposed decoders. In Section 4, the simulation results are illustrated and discussed. Finally, some conclusions are highlighted.

## 2. Preliminaries

In this section, we briefly introduce the polar coding method and existing decoders related to this paper for $PC(N,K+N_{crc})$. $PC(N,K+N_{crc})$ refers to a polar code of code length *N*, CRC length $N_{crc}$, information bit length *K*, and code rate R=K/N.

### 2.1. Polar Encoding Method

Reference [1] defines the polar encoding method as follows:

(1) $$x_{1}^{N}=u_{1}^{N}B_{N}F^{⊗n},$$

where $x_{1}^{N}=\left(x_{1},x_{2},\ldots ,x_{N}\right)$ refers to the encoded vector. $u_{1}^{N}=\left(u_{1},u_{2},\ldots ,u_{N}\right)$ represents the encoding vector that consists of two subjects: $A^{c}$ and A. $A^{c}$ consists of all frozen bits. A consists of all non-frozen bits. $F=\left[\begin{array}{cc} 1 & 0\\ 1 & 1 \end{array}\right]$ and ⊗ refers to the Kronecker product. $B_{N}$ is a bit-reversal permutation matrix. $n=log_{2}N$.

### 2.2. CA-SCL Decoder

The CA-SCL decoder [5,6] is an improved decoder based on the SC decoder and utilizes CRC as well as a list of multiple candidate paths to improve the FER performance of the SC decoder with a single candidate path. The list size of the CA-SCL decoder is *L*. Meanwhile, a simplified path metric (PM) [7] is adopted by the CA-SCL decoder to distinguish the correct path ion the list. PM can be computed using the following equation:

(2) $${PM}_{c}^{\left(i\right)}=\left\{\begin{array}{cc} {PM}_{c}^{\left(i-1\right)}, & \mathrm{if}\;{\hat{u}}_{i}\left[l\right]=\delta \left(\lambda_{N}^{\left(i\right)}\left[l\right]\right);\\ {PM}_{c}^{\left(i-1\right)}+\left|\lambda_{N}^{\left(i\right)}\left[l\right]\right|, & \mathrm{otherwise}. \end{array}\right.$$

where ${PM}_{l}^{\left(i\right)}$ represents the PM value of the *i*th bit in the *l*th candidate path, 0<l<=L and ${PM}_{l}^{\left(0\right)}=0$. $\lambda_{N}^{\left(i\right)}\left[l\right]$ is the log-likelihood ratio (LLR) value of the *i*th bit in the *l*th candidate path. $\delta \left(x\right)=\frac{1}{2}\left(1-sign\left(x\right)\right)$. In addition, we define $A^{\prime }$ as a set consisting of the first $log_{2}L$ of non-frozen bits.

When decoding the *i*th bit and $i\in A^{\prime }$, the CA-SCL decoder utilizes a list $\rfloor_{best}^{\left(i\right)}$ to reserve all candidate paths. When decoding the *i*th bit and $i\in A\setminus A^{\prime }$, the *L* paths in $\rfloor_{best}^{\left(i-1\right)}$ are expanded to 2L sub-paths, which forms an expanded list $\rfloor^{\left(i\right)}$. Then, $\rfloor_{best}^{\left(i\right)}$ can be achieved by selecting *L* paths with a smaller PM from $\rfloor^{\left(i\right)}$. When decoding the *i*th bit and $i\in A^{c}$, the *L* paths in $\rfloor_{best}^{\left(i\right)}$ are not expanded, and the values of the *i*th bit in all candidate paths are 0 by default.

After all bits are decoded, the CA-SCL decoding algorithm performs CRC on the paths in the $\rfloor_{best}^{\left(N\right)}$. If CRC succeeds, the CA-SCL decoder will output the candidate path with the smallest PM from among the candidate paths that passed the CRC check. If not, the CA-SCL decoder will output the candidate path with the smallest PM in $\rfloor_{best}^{\left(N\right)}$.

### 2.3. SCL-Flip Decoder, SCL-SP Decoder, and ADOSPL Decoder

To further improve the performance of the CA-SCL decoder, Ref. [18] proposed the first list flip decoder, named SCL-BF decoder. This decoder brings the flip operation idea to the CA-SCL decoder for the first time, thus allowing it to have better performance than the CA-SCL decoder with the same list size through more decoding attempts with flip operations. Obviously, the performance gain is closely related to the list flip operation itself and the decision with regard to list flip positions. To further improve the performance of the SCL-BF decoder, Ref. [21] proposed the SCL-SP decoder to optimize the list flip operation, and [20] designed the SCL-Flip decoder to optimize the decision of flip positions.

**Figure 1:**
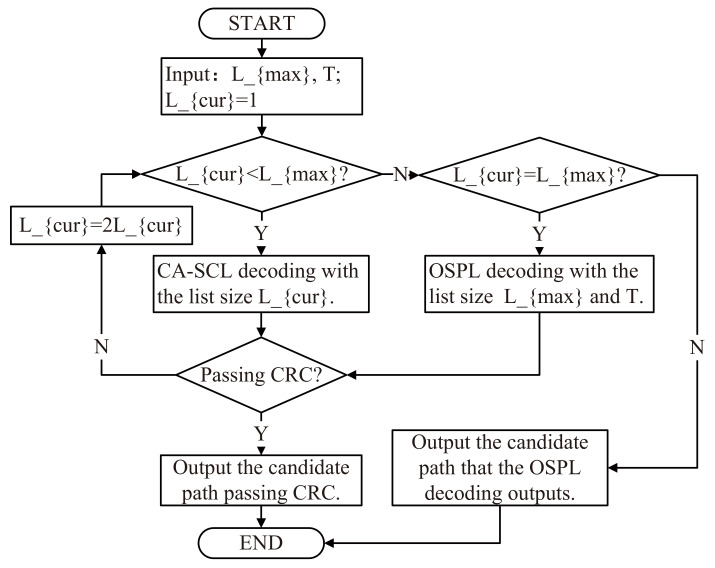
The flowchart of the ADOSPL decoder.

The SCL-SP decoder uses a new list flip operation called the shift-pruning operation to replace the original flip operation; it achieves better performance than the SCL-BF decoder with the same list size. The new list flip operation refers to $\rfloor_{best}^{\left(i\right)}$ and can be achieved by selecting *L* paths with a bigger PM from $\rfloor^{\left(i\right)}$ when the *i*th bit channel is a list flip position. Furthermore, to improve the performance of the SCL-SP decoder, Ref. [22] optimized the flip order of the SCL-SP decoder, introduced the adaptive list, and proposed a new list flip decoder called the adaptive-ordered shifted-pruning list (ADOSPL) decoder. The flowchart of the ADOSPL decoder is shown in Figure 1. When $L_{cur}<L_{max}$, the ADOSPL decoder performs a CA-SCL decoding operation with the list size $L_{cur}$ to obtain the estimated code word. When $L_{cur}=L_{max}$, the ADOSPL decoder executes the OSPL decoder (a list flip decoder proposed by [22]) with a list size of $L_{max}$ to obtain the estimated code word. Since the OSPL decoder has little correlation with this paper, we will not provide more details about this algorithm. The termination condition of the ADOSPL decoder is: either a candidate path passing the CRC is found in a decoding attempt, or $T+1+Log_{2}\left(L\right)$ decoding attempts have been performed. *T* is the maximum number of additional decoding attempts with list flip operations.

The SCL-Flip decoder employs a flip metric to optimize the decision of the SCL-BF decoder with regard to list flip positions. This flip metric satisfies the following equation:

(3) $$E_{\alpha }^{\left(i\right)}=ln\frac{\sum_{l=1}^{c}e^{-PM_{l}^{\left(i\right)}}}{{\left(\sum_{l=1}^{c}e^{-PM_{l+L}^{\left(i\right)}}\right)}^{\alpha }},$$

where $E_{\alpha }^{\left(i\right)}$ denotes the flip metric $E_{\alpha }$ value of the *i*th bit, and α is a coefficient to compensate for the biased estimation due to the error propagation. A lower $E_{\alpha }$ means that mistakes are more likely to occur. Because of this, the SCLFlip decoder prioritizes the flipping of information bits with lower $E_{\alpha }$ value.

Although these list flip decoders can achieve better performance than the CA-SCL decoder to some extent, they cannot correct high-order errors efficiently, which limits the performance gain brought by the list flip operations.

### 2.4. D-SCLF Decoder

To further improve the performance of list flip decoders, the D-SCLF decoder [24] applies a new flip metric to approximate the probability of $\varepsilon^{\left(i\right)}$ occurring in a CA-SCL decoding process. $\varepsilon^{\left(i\right)}=\left\{u_{1}^{i-1}\in \rfloor_{best}^{\left(i-1\right)},u_{1}^{i}\notin \rfloor_{best}^{\left(i\right)}\right\}$ represents the event where the first mistake happened in the *i*th bit. Based on the new flip metric, D-SCLF extends this flip metric to approximate the probability of high-order errors and utilizes a dynamic list of flip sets S to reserve the potential location of high-order errors. The extended flip metric in [24] can be achieved by using the following calculation:

(4) $$\begin{array}{cc} M_{\beta }^{\left(i\right)} & =-\frac{1}{\beta }ln(P\left(\varepsilon^{\left(i\right)}|y,S_{t}\right)\\ \; & =-\frac{1}{\beta }ln\left(P_{e}^{\left(i\right)}\right)-\frac{1}{\beta }\sum_{k\in S_{t}}^{}ln\left(P_{e}^{\left(k\right)}\right)-\frac{1}{\beta }\sum_{k<i,k\in \left\{A\setminus A^{\prime }\right\}\setminus S_{t}}^{}ln\left(1-P_{e}^{\left(k\right)}\right)\\ \; & =E_{1}^{\left(i\right)}+f_{\beta }\left(E_{1}^{\left(i\right)}\right)+\sum_{k\in S_{t}}^{}E_{1}^{\left(k\right)}+\sum_{k\in S_{t}}^{}f_{\beta }\left(E_{1}^{\left(k\right)}\right)+\sum_{k<i,k\in \left\{A\setminus A^{\prime }\right\}\setminus S_{t}}^{}f_{\beta }\left(E_{1}^{\left(k\right)}\right)\\ \; & =E_{1}^{\left(i\right)}+\sum_{k\in S_{t}}^{}E_{1}^{\left(k\right)}+\sum_{k<=i,k\in A\setminus A^{\prime }}^{}f_{\beta }\left(E_{1}^{\left(k\right)}\right), \end{array}$$

where β is also a compensated coefficient similar to α, $S_{t}$ is a flip set that records all flip indices for the *t*th additional decoding attempt, $f_{\beta }\left(x\right)=\frac{1}{\beta }ln\left(1+e^{-\beta x}\right)$ and $E_{1}^{\left(i\right)}=E_{\alpha =1}^{\left(i\right)}$. It is worth noting that $S_{t}$ is a subset of S, and $S=S_{1},S_{2},\ldots ,S_{T}$ has a constant size of *T* but is updated after a failure in decoding attempts. $P(\varepsilon^{\left(i\right)}|y,S_{t})$ refers to the probability that $\varepsilon^{\left(i\right)}$ occurs with the flip set $S_{t}$ [24], and it satisfies the following equation:

(5) $$P\left(\varepsilon^{\left(i\right)}|y,S_{t}\right)=P_{e}^{\left(i\right)}\cdot \prod_{k\in S_{t}}^{}P_{e}^{\left(k\right)}\cdot \prod_{k<i,k\in \left\{A\setminus A^{\prime }\right\}\setminus S_{t}}^{}\left(1-P_{e}^{\left(k\right)}\right),$$

where $P_{e}^{\left(i\right)}=\frac{1}{1+e^{\beta \cdot E_{1}^{\left(i\right)}}}$.

Similar to the SCL-Flip decoder, the D-SCLF decoder prioritizes flipping information bits set with a lower $M_{\beta }$ value computed by (4). However, compared to the flip metric of the SCL-Flip decoder, this flip metric $M_{\beta }$ introduces many logarithmic and exponential operations, especially when the D-SCLF decoder is applied to higher-order error correction. This will limit the application value of this decoder.

## 3. ALF Decoder

In this section, we describe our decoder based on two points: (1) the design of the simplified flip metric and (2) the adoption of the adaptive list. Furthermore, we describe the details of our decoder in Section 3.3.

### 3.1. Design of the Simplified Flip Metric

As mentioned above, the D-SCLF decoder creates a new flip metric to correct high-order errors, and it can improve the error-correction performance of the SCL-Flip decoder. However, we noticed that the new flip metric $M_{\beta }$ introduces an new $f_{\beta }\left(\cdot \right)$ function, including additional logarithmic and exponential operations, while the number of these operations increases exponentially with an increase in the number of unfrozen bits and the order of error correction. It should be noted that the order of error correction refers to the maximum number of flip operations in one decoding attempt. For better understanding, we describe below how the number of operations varies under different conditions.

If there is no extra memory to keep the previous $f_{\beta }\left(\cdot \right)$ value, and the error-correction order of the D-SCLF decoder is 1 (called D-SCLF1 in the following), the D-SCLF1 decoder needs to repeatedly perform the $f_{\beta }\left(\cdot \right)$ function of all previous non-frozen bits after a new non-frozen bit is decoded. Therefore, in this case, the DSCLF-1 decoder executes the $f_{\beta }\left(\cdot \right)$ function $N_{f}^{\left(1\right)}$ times, where $N_{f}^{\left(1\right)}=1+2+\cdots +\left(K+N_{crc}-log_{2}L\right)=\frac{\left(K+N_{crc}-log_{2}L\right)\times \left(K+N_{crc}-log_{2}L+1\right)}{2}$. *L* is the list size of the D-SCLF1 decoder. $N_{f}^{\left(x\right)}$ is the total number of times the $f_{\beta }\left(\cdot \right)$ function is executed, which is required by the D-SCLF*x* decoder to decode one frame.

If all $f_{\beta }\left(\cdot \right)$ values are stored and the D-SCLF1 decoder is still adopted, $N_{f}^{\left(1\right)}=K+N_{crc}-log_{2}L$. However, even if $f_{\beta }\left(\cdot \right)$ values are stored in order to reduce $N_{f}^{\left(1\right)}$, as long as the order of error correction is more than 1, the D-SCLF decoder will execute more $f_{\beta }\left(\cdot \right)$ functions. For example, if D-SCLF2 wants to correct the second-order error, it must generate a set containing two flip positions, assuming that the new flip set is $S_{x}$ and $S_{x}=S_{t}\cup \left\{j\right\}$, where $j>i_{t}$ and x>t. $S_{t}$ refers to the flip set in the *t*th additional attempt and contains only one flip position. $i_{t}$ is the last element in $S_{t}$. According to Equation (4), the D-SCLF2 decoder does not only need more memory to store new $E_{\alpha =1}$ values and $f_{\beta }\left(\cdot \right)$ values updated by the *t*th decoding attempt, but it also needs to calculate at least $K+N_{crc}-i_{t}$ times of the $f_{\beta }\left(\cdot \right)$ function based on these new stored values. Moreover, if the $S_{t}$-based $S_{x}$ cannot help the D-SCLF2 decoder to find the correct path, D-SCLF2 will continue to perform new $f_{\beta }\left(\cdot \right)$ functions based on other first-order flip sets. Hence, $N_{f}^{\left(2\right)}$ is significantly larger than $N_{f}^{\left(1\right)}$, and thus $N_{f}^{\left(x\right)}$ will increase exponentially with *x* increases.

Therefore, to expand the application value of the D-SCLF decoder, it is necessary to design a simplified flip metric that can effectively reduce the number of exponent and logarithmic operations included by the $f_{\beta }\left(\cdot \right)$ function without losing error-correction performance.

Inspired by the simplification of the decoder of turbo codes in [25] and the simplification of the flip metric in the DSCFlip decoder of polar codes in [16], we naturally thought of using a similar method to simplify the $f_{\beta }\left(\cdot \right)$ function in the D-SCLF decoder. However, we noticed that the original simplified methods in [16,25] did not provide directions that were clear enough, so this paper attempts to design a simplified function with actionable steps to simplify the D-SCLF decoder. Since multiple straight lines can approximate a curve, and different straight lines essentially correspond to different combinations of one addition and one multiplication, this paper proposes a simplified function called the $f_{\beta }^{line}\left(\cdot \right)$ function, which utilizes two straight line segments to approximate the curve represented by the $f_{\beta }\left(\cdot \right)$ function in the D-SCLF decoder.

**Figure 2:**
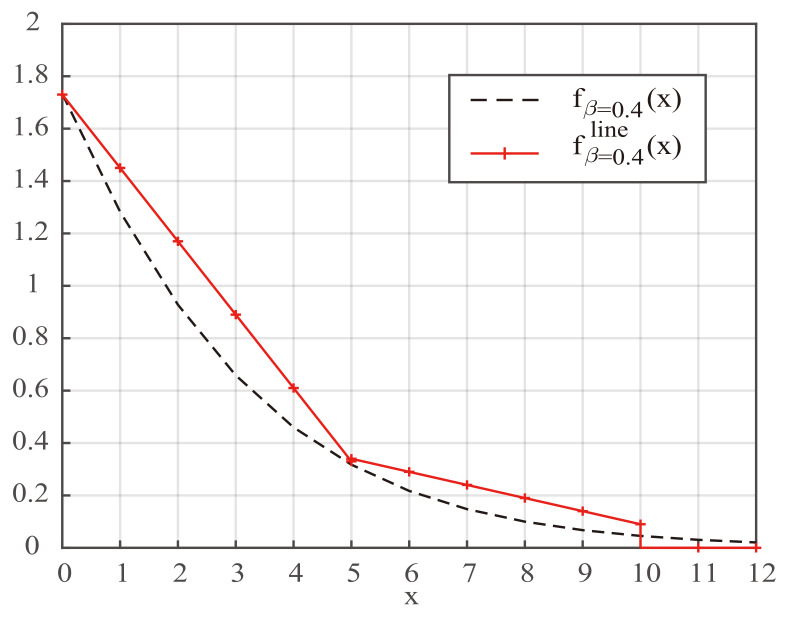
Comparison of $f_{\beta =0.4}\left(x\right)$ and $f_{\beta =0.4}^{line}\left(x\right)$, with z=5.

To illustrate our simplified function more clearly, we created the graph in Figure 2. In Figure 2, the black dashed line indicates the $f_{\beta }\left(\cdot \right)$ function, and the red line represents our simplified function. Evidently, our simplified function is mainly composed of two straight line segments, and these two straight line segments are generated based on three points on the black dashed line. Since the $E_{\alpha =1}$ value in Equation (4) satisfies $E_{\alpha =1}>0$, we selected (0,$f_{\beta }\left(0\right)$) as the first point. Based on the experience provided by the existing simplification, we selected (10,$f_{\beta }\left(10\right)$) as the third point. Finally, we selected (*z*,$f_{\beta }\left(z\right)$) as the second point, where *z* is a positive integer and 1≤z≤10. Therefore, the simplified function $f_{\beta }^{line}\left(\cdot \right)$ satisfies the following equation:

(6) $$f_{\beta }^{line}\left(x\right)=\left\{\begin{array}{cc} c_{1}x+f_{\beta }\left(0\right), & \mathrm{if}\;x\geq 0\;\mathrm{and}\;x\leq z;\\ c_{2}x+c_{3}, & \mathrm{if}\;x>z\;\mathrm{and}\;x\leq 10;\\ 0, & others. \end{array}\right.$$

where $c_{1}=\frac{f_{\beta }\left(z\right)-f_{\beta }\left(0\right)}{z}$, $c_{2}=\frac{f_{\beta }\left(z\right)-f_{\beta }\left(10\right)}{z-10}$, and $c_{3}=f_{\beta }\left(z\right)-z\times \frac{f_{\beta }\left(z\right)-f_{\beta }\left(10\right)}{z-10}$. Additionally, $c_{1}$, $c_{2}$, and $c_{3}$ in this paper generally retain two decimal places. In particular, when z=10,

(7) $$f_{\beta }^{line}\left(x\right)=\left\{\begin{array}{cc} c_{1}x+f_{\beta }\left(0\right), & \mathrm{if}\;x\geq 0\;\mathrm{and}\;x\leq 10;\\ 0, & others. \end{array}\right.$$

It is clear that we used a combination of one multiplication and one addition to replace the multiplication, addition, exponential, and logarithmic operations included by the original $f_{\beta }\left(x\right)=\frac{1}{\beta }ln\left(1+e^{-\beta x}\right)$. Furthermore, Ref. [24] demonstrated through simulation that outstanding performance can be reached with a fixed β of 0.4, so this paper uses a default β value of 0.4.

**Figure 3:**
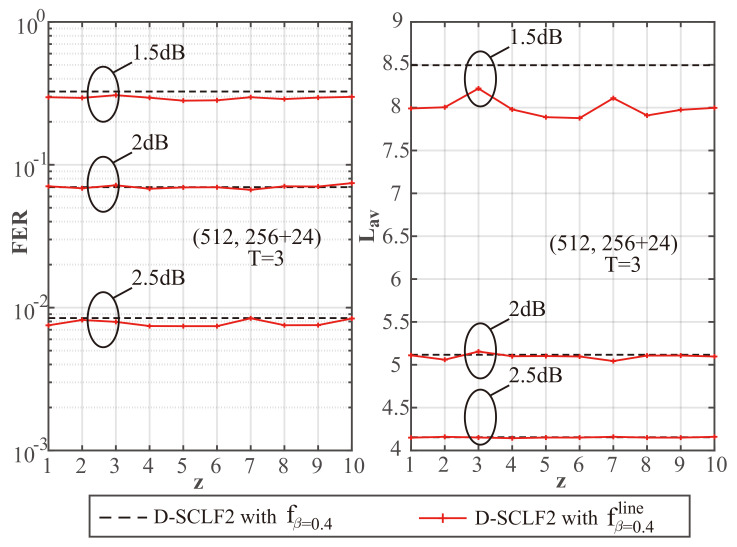
Performance comparison of D-SCLF2 using $f_{\beta =0.4}$ and D-SCLF2 using $f_{\beta =0.4}^{line}$, with L=4, T=3, and different *z* values, for PC(512,256+24).

**Figure 4:**
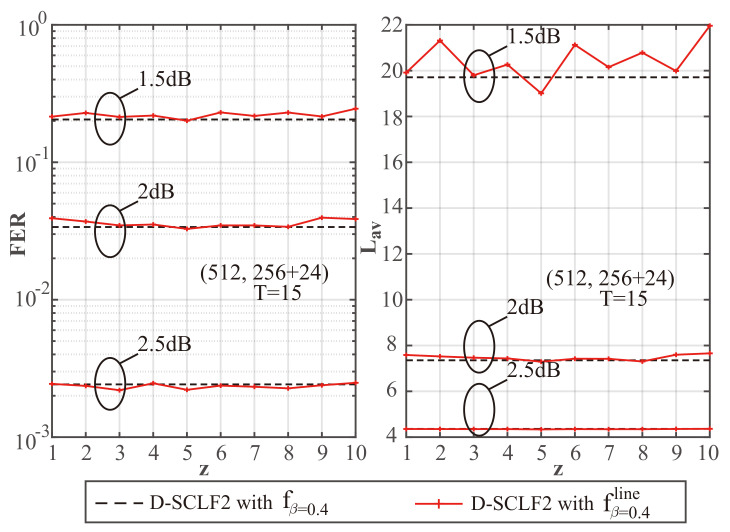
Performance comparison of D-SCLF2 using $f_{\beta =0.4}$ and D-SCLF2 using $f_{\beta =0.4}^{line}$, with L=4, T=15, and different *z* values, for PC(512,256+24).

**Figure 5:**
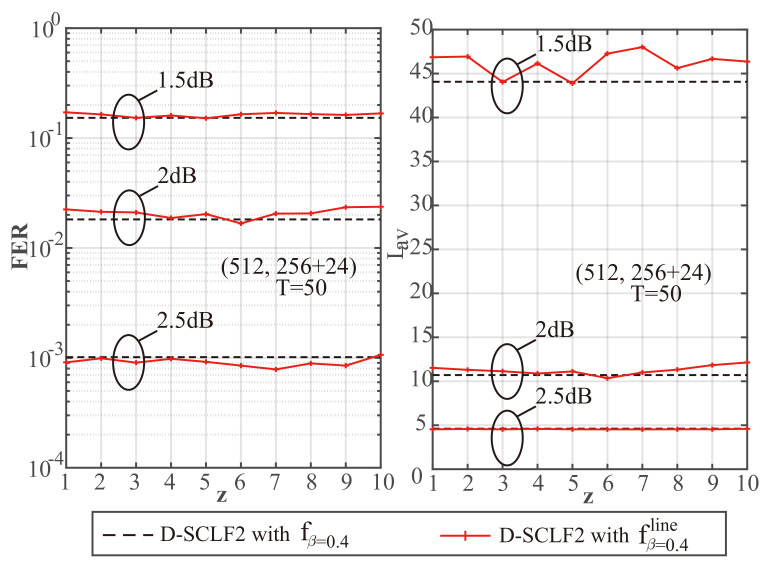
Performance comparison of D-SCLF2 using $f_{\beta =0.4}$ and D-SCLF2 using $f_{\beta =0.4}^{line}$, with L=4, T=50, and different *z* values, for PC(512,256+24).

To explore the impact of different *z* values on performance, we created Figure 3, Figure 4 and Figure 5 to show a comparison of the performance of D-SCLF2 using $f_{\beta =0.4}$ and that of D-SCLF2 using $f_{\beta =0.4}^{line}$, with different *z* values for PC(512,256+24). Note that the D-SCLF2 using $f_{\beta =0.4}$ refers to the D-SCLF2 decoder using the original flip metric, and the D-SCLF2 using $f_{\beta =0.4}^{line}$ refers to the D-SCLF2 decoder using the new simplified flip metric. The list size of these decoders is 4. In these figures, the left sub-graphs represent the FER performance, and the right sub-graphs represent the corresponding average complexity performance. Since the average complexity of the D-SCLF ( or CA-SCL) decoder is $O(\left(T_{av}+1\right)\times LNlogN)$ (or O(LNlogN)), we can use $L_{av}$ to represent the average complexity, where $L_{av}=\left(T_{av}+1\right)\times L$ for the list flip decoder (or $L_{av}=L$ for the CA-SCL decoder ). $T_{av}$ represents the average number of extra decoding attempts. The dashed lines in these figures represent the performance of DSCLF-2 with the original $f_{\beta =0.4}\left(\cdot \right)$ function, and the solid line represents the performance of D-SCLF2 with the new $f_{\beta =0.4}^{line}\left(\cdot \right)$ function. The AWGN channel, BPSK modulation, and the Gaussian Approximation (GA) construction algorithm [26] with a fixed $E_{b}/N_{0}$ of 4 dB are used. The generator polynomial of the 24 CRC bits is $g\left(x\right)=x^{24}+x^{23}+x^{6}+x^{5}+x^{1}+1$.

Figure 3, Figure 4 and Figure 5 show that under the same $E_{b}/N_{0}$, the D-SCLF2 using $f_{\beta =0.4}^{line}$ has a similar FER performance to that of the D-SCLF2 using $f_{\beta =0.4}$. Therefore, our simplified flip metric does not reduce the high-order error correction capability brought by the original flip metric in the D-SCLF decoder, regardless of the *z* value. However, at some *z* values, the D-SCLF2 using $f_{\beta =0.4}^{line}$ may have greater $L_{av}$ than the D-SCLF2 using $f_{\beta =0.4}$. The reason is that the new flip metrics corresponding to some z values are not effective enough, which makes the D-SCLF2 using $f_{\beta =0.4}^{line}$ need more decoding attempts to find the correct path. Thus, a fixed *z* value is required to ensure that the D-SCLF2 using $f_{\beta =0.4}^{line}$ has similar or better performance to the D-SCLF2 using $f_{\beta =0.4}$ under different operating conditions. It is worth mentioning that $L_{av}$ represents the complexity because it is proportional to the number of recursions of the LLR of the polar code, but $L_{av}$ does not count the calculations in the flip metric. Therefore, as long as the FER and $L_{av}$ of the D-SCLF2 using $f_{\beta =0.4}^{line}$ and the D-SCLF2 using $f_{\beta =0.4}$ are similar, our new flip metric is valid.

To better identify the fixed *z* value, we created Table 1, Table 2 and Table 3 to provide more details on the complexity comparison found in Figure 3, Figure 4 and Figure 5. In these tables, $R_{c}$ represents the reduction ratio of the current $L_{av}$ as compared to the $L_{av}$ corresponding to a “no z” situation; a “no z” situation refers to the D-SCLF2 using $f_{\beta =0.4}$, while a “z = x” situation refers to the D-SCLF2 using $f_{\beta =0.4}^{line}$ with the *z* value of *x*. For easier understanding, we use the data in Table 1 as an example. In Table 1, the $L_{av}$ value of the D-SCLF2 using $f_{\beta =0.4}$ is 8.495 when $E_{b}/N_{0}=1.5$ dB, and the corresponding $R_{c}$ satisfies $R_{c}=\left(8.495-8.495\right)/8.495=0\%$. Similarly, the $L_{av}$ value of the D-SCLF2 using $f_{\beta =0.4}^{line}$ with z=1 is 7.991 when $E_{b}/N_{0}=1.5$ dB, and the corresponding $R_{c}$ satisfies $R_{c}=\left(8.495-7.991\right)/8.495=5.94\%$.

Based on the above discussion, a fixed and appropriate *z* value should satisfy two points: (i) The FER performance of the DSCLF-2 using $f_{\beta =0.4}^{line}$ cannot be significantly worse than that of the D-SCLF2 using $f_{\beta =0.4}$; (ii) The $L_{av}$ value of the DSCLF-2 using $f_{\beta =0.4}^{line}$ cannot be significantly greater than that of the D-SCLF2 using $f_{\beta =0.4}$. Therefore, we choose a fixed *z* value based on the above two points and think of the first point as having a higher priority. Finally, we choose a *z* value of 5. Then, we can obtain the following equation:

(8) $$f_{\beta }^{line}\left(x\right)=\left\{\begin{array}{cc} -0.28x+1.72, & \mathrm{if}\;x\geq 0\;\mathrm{and}\;x\leq 5;\\ -0.05x+0.59, & \mathrm{if}\;x\geq 5\;\mathrm{and}\;x\leq 10;\\ 0, & others. \end{array}\right.$$

Thus, our simplified flip metric satisfies the equation below:

(9) $$M_{\beta }^{line}\left(S_{t}⋃\left\{i\right\}\right)=E_{1}^{\left(i\right)}+\sum_{k\in S_{t}}^{}E_{1}^{\left(k\right)}+\sum_{k<=i,k\in A\setminus A^{\prime }}^{}f_{\beta =0.4}^{line}\left(E_{1}^{\left(k\right)}\right),$$

where $i>i_{t}$, and $i_{t}$ is the last element in $S_{t}$. Moreover, we use the more understandable $M_{\beta }^{line}\left(S_{t}⋃\left\{i\right\}\right)$ to represent the flip metric of high-order flip operations. In particular,

(10) $$M_{\beta }^{line}\left(S_{t}\right)=\sum_{k\in S_{t}}^{}E_{1}^{\left(k\right)}+\sum_{k<=i_{t},k\in A\setminus A^{\prime }}^{}f_{\beta =0.4}^{line}\left(E_{1}^{\left(k\right)}\right),$$

and

(11) $$M_{\beta }^{line}\left(\left\{i\right\}\right)=E_{1}^{\left(i\right)}+\sum_{k<=i,k\in A\setminus A^{\prime }}^{}f_{\beta =0.4}^{line}\left(E_{1}^{\left(k\right)}\right).$$

It is worth mentioning that the default value of *z* for our new flip metric is 5, which means that the simplified function remains consistent with Equation (8). Moreover, we will prove in Section 4 that the new flip metric with a *z* value of 5 is valid under different operating conditions.

### 3.2. The Adoption of the Adaptive List

Inspired by [22], we naturally thought of applying an adaptive list to the D-SCLF decoder to reduce its average complexity. Compared with [22], we provide a more in-depth discussion on the reasons for this application as well as more in-depth theoretical analyses of the impact of this application on performance. Furthermore, we verify these theoretical analyses by simulation in Section 4.

Since a list flip decoder is converted into a list decoder when T=0, we use the default value of T>0 to better analyze the combination of the list flip decoder and the adaptive list.

**The negative impact of applying an adaptive list in list flip decoders is lower than that of applying the adaptive list in list decoders.** We denote $L_{av}^{w}$ as the $L_{av}$ for the worst case, and $L_{max}$ as the maximum list size. Then, the $L_{av}^{w}$ of the CA-SCL decoder and the CA-SCL decoder using an adaptive list [8] is $L_{max}$ and $2\times L_{max}-1$, respectively. The $L_{av}^{w}$ of a list flip decoder and the list flip decoder with an adaptive list is $\left(T+1\right)\times L_{max}$ and $\left(T+2\right)\times L_{max}-1$, respectively. Since T>0, we can obtain $\frac{2\times L_{max}-1}{L_{max}}>\frac{\left(T+2\right)\times L_{max}-1}{\left(T+1\right)\times L_{max}}$. Therefore, the negative impact of applying the adaptive list on the list flip decoder is lower. In particular, $\frac{\left(T+2\right)\times L_{max}-1}{\left(T+1\right)\times L_{max}}$ approaches 1 when *T* increases.

**A list flip decoder with an adaptive list (LFD-w) and the same list flip decoder without the adaptive list (LFD-o) have similar error-correction performance.** To illustrate this, we divided all possible decoding processes of an LFD-w into three cases:

- Case 1: The LFD-w can output the path passing CRC when the current list size is $L_{cur}$ and $L_{cur}<L_{max}$;
- Case 2: The LFD-w can output the path passing CRC when the current list size is $L_{cur}$ and $L_{cur}=L_{max}$;
- Case 3: The LFD-w cannot output the path passing CRC.

For case 1, the PM value of the path passing CRC is small enough to always be retained by a small list, which means that the path has a high probability of being retained by a larger list as well. In other words, the LFD-o can find this path with a high probability.

For case 2, the path passing CRC can be found by the LFD-w when $L_{cur}=L_{max}$, which means the correct path can also be found by the LFD-o with a list of list size $L_{max}$.

For case 3, if the path passing CRC cannot be found by the LFD-w, the LFD-o also fails to find the path since the LFD-w includes the LFD-o.

According to the above analyses, the error-correction performance of the LFD-w may be consistent with that of the LFD-o. However, there are two special situations included by the above cases. One is that a small size list ($L<L_{max}$) can preserve the correct path, but a large size list ($L=L_{max}$) may prune the correct path since the PM value of the correct path may be more than that of other candidate paths in this large size list. The other is that a wrong path passing CRC can be preserved by a small size list ($L<L_{max}$) but be removed by a larger size list ($L=L_{max}$). Both of these special situations will affect the error-correction performance of the LFD-w and the LFD-o to a certain extent. Considering that these two cases are relatively rare and that CRC is a reliable verification method, the LFD-w and the LFD-o have similar error-correction performance.

**The introduction of the adaptive list greatly reduces the complexity of the list flip decoders at a high SNR.** We denote $L_{av}^{b}$ as the $L_{av}$ for the best case. Then, the $L_{av}^{b}$ of an LFD-w and an LFD-o is 1 and $L_{max}$, respectively. Therefore, the LFD-w can efficiently reduce the complexity of the LFD-o when $L_{max}>1$ and the SNR is high.

Based on the above analyses, we believe the combination of the adaptive list and the list flip decoder is very suitable. Furthermore, we propose our ALF decoder by adopting the simplified flip metric and an adaptive list; its details are described in Section 3.3.

### 3.3. Details of the ALF Decoder

Algorithm 1 describes the details of our ALF decoder. *T* refers to the maximum of extra decoding attempts with the list flip operation. $L_{max}$ represents the maximum list size. $L_{cur}$ is the current list size. $S=\{S_{1},S_{2},\ldots ,S_{T}\}$ refers to the list of flip sets. Its subset $S_{t}$ is a flip set that records all flip indices for the *t*th additional decoding attempt. It is worth noting that $S_{0}$ is not a subset of S, and $S_{0}=\emptyset$. $M=\{M_{\beta }^{line}\left(S_{1}\right),M_{\beta }^{line}\left(S_{2}\right),\ldots ,M_{\beta }^{line}\left(S_{T}\right)\}$ is the list of flip metric values that satisfies $M_{\beta }^{line}\left(S_{1}\right)<M_{\beta }^{line}\left(S_{2}\right)<\ldots <M_{\beta }^{line}\left(S_{T}\right)$. The initial S and M are both ${\left[0\right]}_{1\times T}$. **SCLDecoding**($S_{t}$, $L_{cur}$) denotes a standard CA-SCL decoding with a list of size $L_{cur}$ during which the flip operation will be performed at the bit indices given in this set $S_{t}$. In particular, **SCLDecoding**(*∅*, $L_{cur}$) denotes a standard CA-SCL decoding without the flip operation. The flip operation refers to the current $\rfloor_{best}^{\left(i\right)}$ consisting of *L* paths with bigger PM from $\rfloor^{\left(i\right)}$. ${\hat{u}}_{1}^{N}$ is the estimated sequence of $u_{1}^{N}$.

When $L_{cur}<L_{max}$, **SCLDecoding**(*∅*, $L_{cur}$) is performed to obtain ${\hat{u}}_{1}^{N}$. If the ${\hat{u}}_{1}^{N}$ passes CRC, ALF decoding is terminated and outputs the current ${\hat{u}}_{1}^{N}$. If not, $L_{cur}$ is updated to $2\times L_{cur}$ and the ALF decoding continues.

When $L_{cur}=L_{max}$, **SCLDecoding**($S_{t}$, $L_{max}$) is performed to obtain ${\hat{u}}_{1}^{N}$. If the ${\hat{u}}_{1}^{N}$ passes CRC, the ALF decoding is terminated and outputs the current ${\hat{u}}_{1}^{N}$. If not, (S, M) is updated by performing the **UpdateFlipList**(·) function, *t* is updated to t+1, and the ALF decoding continues.
**Algorithm 1:** ALF decoder
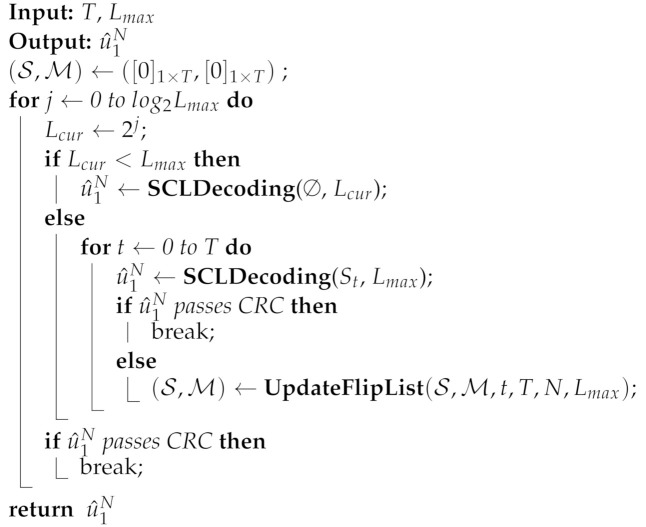


Algorithm 2 describes the update process of (S,M).

If t=0, S will consist of *T* indexes of non-frozen bits with smaller $M_{\beta }^{line}$, and these non-frozen bits are in $\left\{A\setminus A^{\prime }\right\}$. After S is updated, M will store the flip metrics corresponding to the flip sets in S, which satisfies $M=\{M_{\beta }^{line}\left(S_{1}\right),M_{\beta }^{line}\left(S_{2}\right),\ldots ,M_{\beta }^{line}\left(S_{T}\right)\}$, and $M_{\beta }^{line}\left(S_{1}\right)<M_{\beta }^{line}\left(S_{2}\right)<\ldots <M_{\beta }^{line}\left(S_{T}\right)$. It is worth noting that $S_{t}$ refers to the *t*th element in S.

If 0<t<T, S will be updated by inserting new flip sets $\left\{S_{t}\cup \left\{j\right\}\right\}$ when $j>i_{t}$, $j\in \{A\setminus A^{\prime }\}$, $M_{\beta }^{line}\left(S_{t}\cup \left\{j\right\}\right)<M_{\beta }^{line}\left(S_{T}\right)$. After S is updated, M will store the new flip metrics corresponding to the updated S, which satisfies $M=\{\ldots ,M_{\beta }^{line}\left(S_{t}\right),\ldots ,M_{\beta }^{line}\left(S_{t}\cup \left\{j\right\}\right),\ldots ,M_{\beta }^{line}\left(S_{T-1}\right)\}$, and $M_{\beta }^{line}\left(S_{1}\right)<\ldots <M_{\beta }^{line}\left(S_{t}\right)<\ldots <M_{\beta }^{line}\left(S_{t}\cup \left\{j\right\}\right)<\ldots <M_{\beta }^{line}\left(S_{T-1}\right)$. It is worth noting that the new $S_{T}$ in the updated S is the $S_{T-1}$ in the S before being updated.

If t⩾T, S and M will not be updated.
**Algorithm 2:UpdateFlipList**()
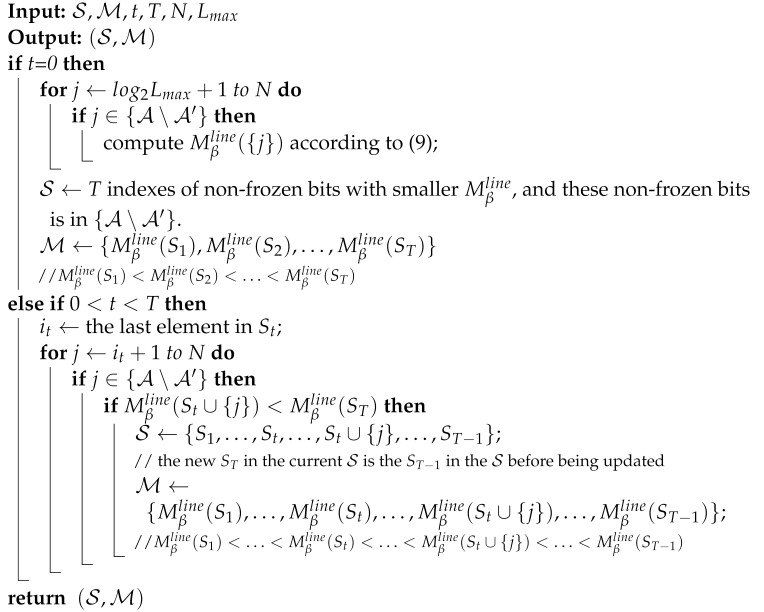


## 4. Simulation Results and Discussions

In this section, we compare the performance of the ALF decoder, the D-SCLF decoder, and the CA-SCL decoder. The default value of *z* for our new flip metric is 5. The order of the list flip decoders is 2 for comparing the high-order error-correction capacity. The AWGN channel, BPSK modulation, and the GA construction algorithm with a fixed $E_{b}/N_{0}$ of 4 dB are used. The generator polynomial of the 24 CRC bits is $g\left(x\right)=x^{24}+x^{23}+x^{6}+x^{5}+x^{1}+1$.

**Figure 6:**
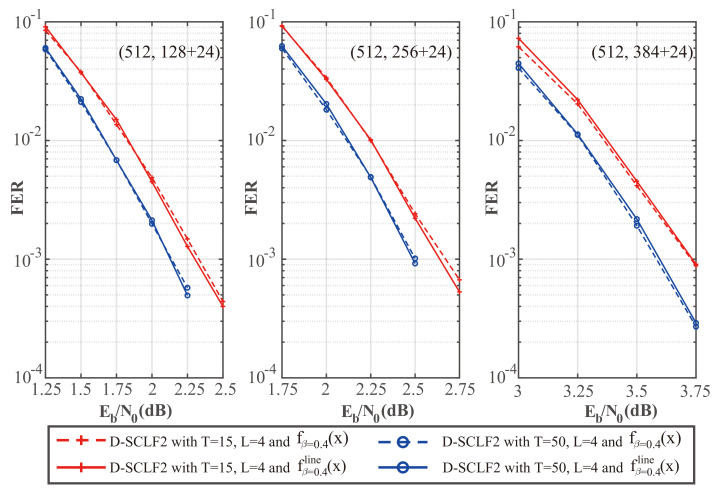
Comparison of the FER performance of the D-SCLF2 with the original flip metric and the D-SCLF2 with the simplified flip metric, for PC(512,128+24), PC(512,256+24), and PC(512,384+24).

**Figure 7:**
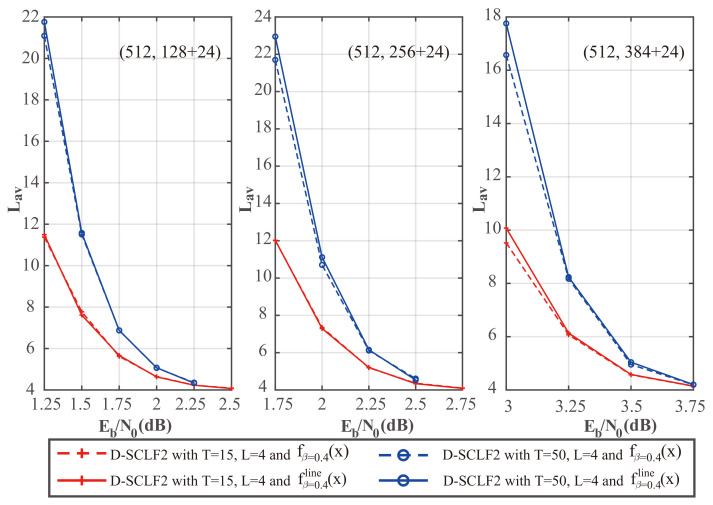
Comparison of the average complexity performance of the D-SCLF2 with the original flip metric and the D-SCLF2 with the simplified flip metric, for PC(512,128+24), PC(512,256+24), and PC(512,384+24).

To further verify whether our simplified flip metric is still valid with different code rates, we plotted Figure 6 and Figure 7, which show the comparison of the performance of the D-SCLF2 decoder using the original flip metric and the D-SCLF2 decoder using the simplified flip metric for different code rates. In these figures, solid lines represent the performance of the D-SCLF2 decoder using our simplified flip metric, and dotted lines represent the performance of the D-SCLF2 decoder using the original flip metric. $L_{max}=4$.

We noticed that these two kinds of curves under the same parameters almost overlap, which means our simplified flip metric is still valid for different code rates and *T* values. Moreover, the D-SCLF2 decoder using the simplified flip metric has better FER performance than the D-SCLF2 decoder using the original flip metric in some nodes. The reason is that the original flip metric is only an approximation of the occurrence probability of high-order errors, and the accuracy of this flip metric has room for further optimization.

**Figure 8:**
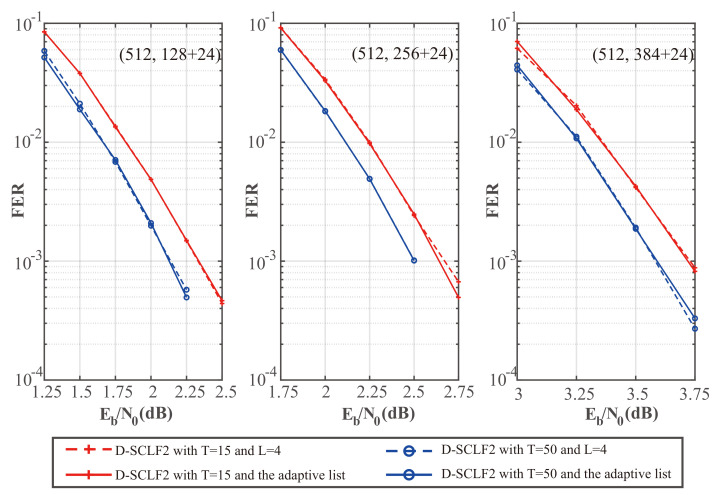
Comparison of the FER performance of the D-SCLF2 without an adaptive list and the D-SCLF2 with an adaptive list, for PC(512,128+24), PC(512,256+24), and PC(512,384+24).

**Figure 9:**
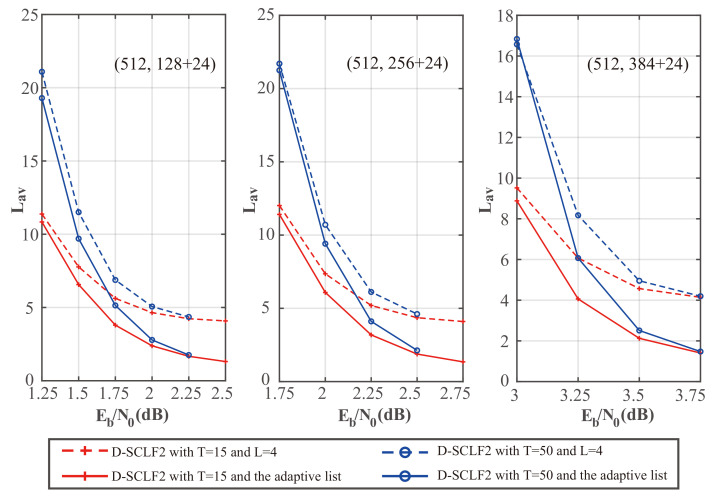
Comparison of the average complexity performance of the D-SCLF2 without an adaptive list and the D-SCLF2 with an adaptive list, for PC(512,128+24), PC(512,256+24), and PC(512,384+24).

To verify the theoretical analyses of the adaptive list in Section 3.2, we drew Figure 8 and Figure 9, which show the comparison of the performance of the original D-SCLF2 decoder and the D-SCLF2 decoder using the adaptive list. In these figures, solid lines represent the performance of the D-SCLF2 decoder using the adaptive list, and dotted lines represent the performance of the original D-SCLF2 decoder. It is worth noting that the Lmax of the D-SCLF2 decoder using the adaptive list is equal to the list size of the original D-SCLF2 decoder; $L_{max}=4$. With the same code rate and *T*, we observed that the FER performance curves of the two decoders almost overlap, which means these two decoders have similar error-correction performance. Moreover, it can be seen in Figure 9 that the average complexity of the D-SCLF2 decoder using the adaptive list is significantly lower than that of the original D-SCLF2 decoder at $FER=10^{-3}$, which means that the introduction of the adaptive list greatly reduces the complexity of the D-SCLF2 decoder at a high SNR. Therefore, the simulation results are consistent with the theoretical analyses in Section 3.2.

**Figure 10:**
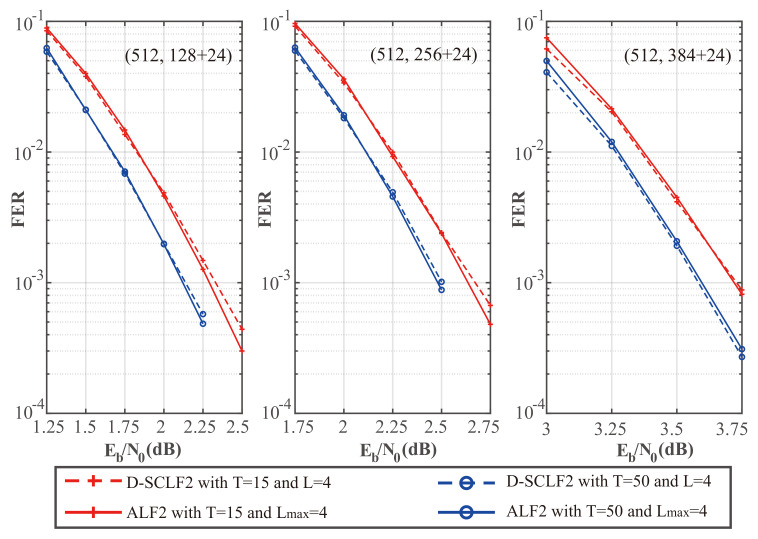
Comparison of the FER performance of the D-SCLF2 and the ALF2, for PC(512,128+24), PC(512,256+24), and PC(512,384+24).

**Figure 11:**
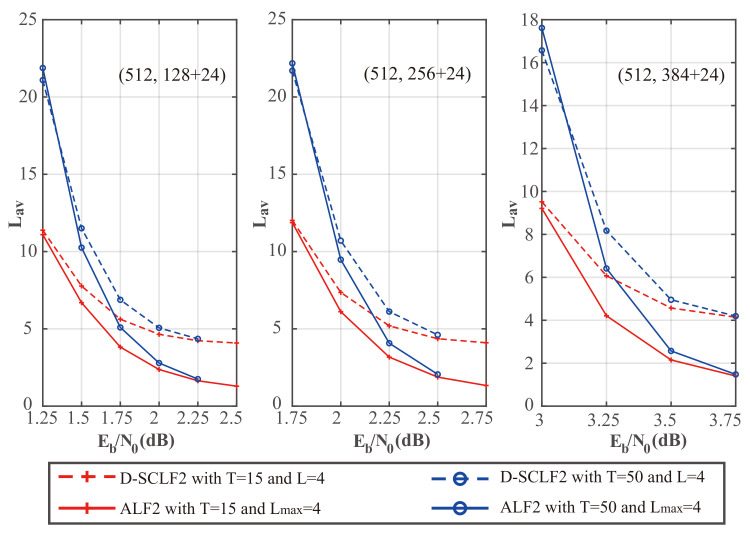
Comparison of the average complexity performance of the D-SCLF2 and the ALF2, for PC(512,128+24), PC(512,256+24), and PC(512,384+24).

To compare the performance of our ALF2 decoder and the D-SCLF decoder, we drew Figure 10 and Figure 11, which show the performance comparison of the original D-SCLF2 decoder and our ALF2 decoder for different code rates. In these figures, solid lines represent the performance of our ALF2 decoder, and dotted lines represent the performance of the original D-SCLF2 decoder.

Figure 10 shows that our ALF2 has an FER performance similar to that of the D-SCLF2 decoder with the same code rate and *T*. Moreover, the ALF2 decoder has better FER performance than the D-SCLF2 decoder in some nodes since the accuracy of the original flip metric has room for further optimization.

Figure 11 shows a comparison of the average complexity performance of the different decoders in Figure 10.

We noticed that as $E_{b}/N_{0}$ is increased, the average complexity of the ALF2 decoder and the DSCLF2 decoder all decrease regardless of the code rate and *T* value. In all cases, the average complexity of our ALF2 decoder always decreases faster than the D-SCLF2 decoder. In particular, when T=15 and $E_{b}/N_{0}=2.5dB$, our ALF2 decoder can reduce the average complexity of the D-SCLF2 decoder by 68.35% without losing error-correction performance.

To show the complexity performance more clearly, we created Table 4, Table 5 and Table 6, which show the details of the comparison of the average complexity performance in Figure 11.

**Figure 12:**
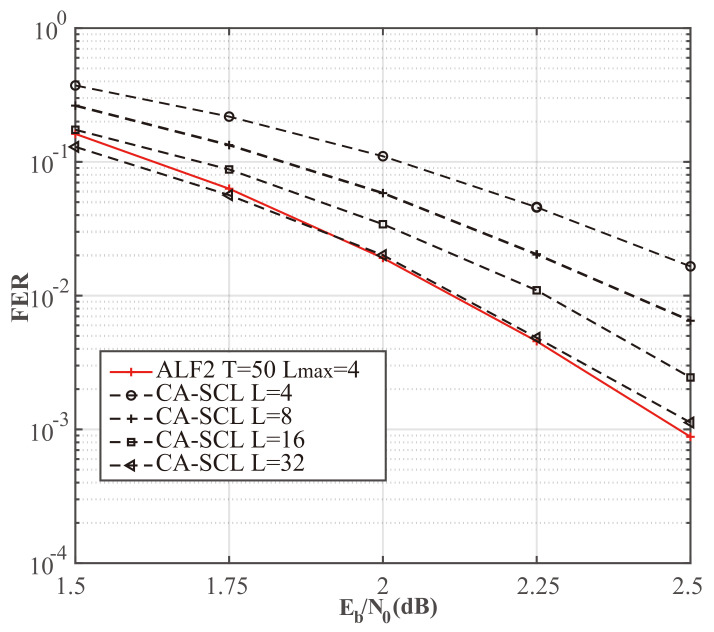
Comparison of the FER performance of the CA-SCL and ALF2, for PC(512,256+24).

Table 4, Table 5 and Table 6 show that the average complexity of our ALF decoder is significantly lower than that of the D-SCLF2 decoder in the $E_{b}/N_{0}$ range corresponding to $FER\in [10^{-2},10^{-3}]$. However, at low $E_{b}/N_{0}$, the complexity of ALF2 may be higher than that of the D-SCLF2 decoder due to the negative impact of the adaptive list discussed in Section 3.2. However, the high complexity at low $E_{b}/N_{0}$ is a problem faced by all list flip decoders (or list decoders with extra decoding attempts). Therefore, in a channel with generally large noise, it is more appropriate to directly use the list decoder with the same error-correction performance. For example, as shown in Figure 12, we can use the decoder of the CA-SCL decoder (L=32) to replace the ALF2 decoder ($L_{max}=4$, T=50) or the D-SCLF2 decoder ($L_{max}=4$, T=50) when the channel noise is generally large. That is to say, in the scenario where there is no memory limitation and the channel noise is generally large, we can replace the list flip decoder with a list decoder. For other scenarios, our decoder is a suitable choice due to its significantly lower complexity and smaller list size.

**Figure 13:**
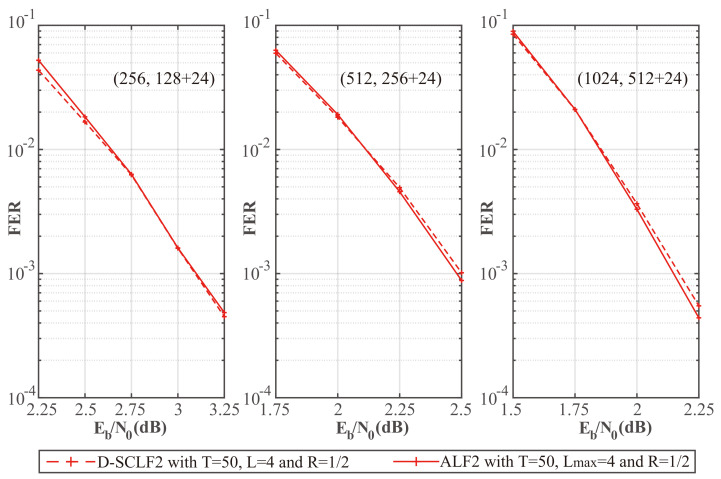
Comparison of the FER performance of the D-SCLF2 and the ALF2, for PC(256,128+24), PC(512,256+24), and PC(1024,512+24).

**Figure 14:**
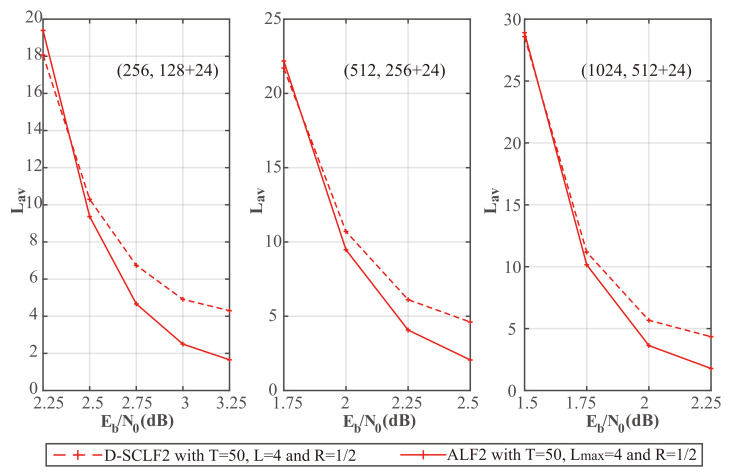
Comparison of the average complexity performance of the D-SCLF2 and the ALF2, for PC(256,128+24), PC(512,256+24), and PC(1024,512+24).

To further verify the advantages of our algorithm, we created Figure 13 and Figure 14, which show the performance comparison of the original D-SCLF2 decoder and our ALF2 decoder for different code lengths. In these figures, solid lines represent the performance of our ALF2 decoder, and dotted lines represent the performance of the original D-SCLF2 decoder.

Figure 13 shows that our ALF2 decoder has an FER performance similar to that of the D-SCLF2 decoder with the same code length. Furthermore, the ALF2 decoder also has better FER performance than the D-SCLF2 decoder in some nodes, such as the phenomenon in Figure 10, since the accuracy of the original flip metric has room for further optimization.

Figure 14 shows the average complexity performance comparison of the different decoders in Figure 13. We noticed that as $E_{b}/N_{0}$ is increased, the average complexity of the ALF2 decoder and the DSCLF2 decoder all decrease, regardless of code lengths. In all cases, the average complexity of our ALF2 decoder always decreases faster than the D-SCLF2 decoder.

Based on the above simulation results, we can conclude that in the practical FER range and in a wide range of code lengths and code rates, our ALF decoder has a significantly lower average complexity than the D-SCLF decoder with a similar error-correction performance. In particular, the new flip metric employed by our ALF decoder can greatly reduce the nonlinear operations introduced by the original flip metric of the D-SCLF decoder without affecting the error correction performance. Moreover, the *z* value of the new flip metric is fixed, and the value is 5.

## 5. Conclusions

To further develop the list flip decoder of polar codes, a new ALF decoder with high-order error correction capability was designed in this paper. Although an existing D-SCLF decoder can achieve high-order error correction capabilities by employing a flip metric, this flip metric introduces new exponential and logarithmic operations. However, the number of these operations increases exponentially as the number of non-frozen bits and the order of error correction increase. To overcome this problem, we designed a new, simplified flip metric, which replaces the logarithmic and exponential operations in the original flip metric with multiplication and addition operations. Simulation results prove that the new flip metric does not reduce the error-correction performance of the existing D-SCLF decoder. In addition, we adopted the adaptive list to further reduce the complexity based on in-depth analyses of the combination of the adaptive list and the list flip decoder. Simulation results show that our ALF decoder can effectively reduce the average complexity (energy) of the existing D-SCLF decoder without losing error-correction performance.

To further exploit the performance potential of list flip decoders, a more accurate flip metric for high-order error correction is worth exploring in future research work.

## Figures and Tables

**Table 1:** Comparison of the average complexity performance of D-SCLF2 using $f_{\beta =0.4}$ and D-SCLF2 using $f_{\beta =0.4}^{line}$, with L=4, T=3, and different *z* values, for PC(512,256+24).

T = 3						
	1.5 dB		2.0 dB		2.5 dB	
	Lav	Rc	Lav	Rc	Lav	Rc
no z	8.495	0.00%	5.117	0.00%	4.154	0.00%
z = 1	7.991	5.94%	5.110	0.13%	4.150	0.11%
z = 2	8.004	5.77%	5.059	1.12%	4.159	−0.12%
z = 3	8.224	3.20%	5.154	−0.74%	4.151	0.08%
z = 4	7.979	6.08%	5.099	0.34%	4.143	**0.27%**
z = 5	7.889	7.13%	5.102	0.29%	4.151	0.08%
z = 6	7.878	**7.27%**	5.096	0.40%	4.151	0.09%
z = 7	8.111	4.53%	5.043	**1.44%**	4.160	−0.12%
z = 8	7.909	6.90%	5.107	0.19%	4.150	0.10%
z = 9	7.974	6.13%	5.108	0.18%	4.150	0.10%
z = 10	7.998	5.85%	5.096	0.39%	4.159	−0.12%

**Table 2:** Comparison of the average complexity performance of D-SCLF2 using $f_{\beta =0.4}$ and D-SCLF2 using $f_{\beta =0.4}^{line}$, with L=4, T=15, and different *z* values, for PC(512,256+24).

T = 15						
	1.5 dB		2.0 dB		2.5 dB	
	Lav	Rc	Lav	Rc	Lav	Rc
no z	19.710	0.00%	7.355	0.00%	4.360	0.00%
z = 1	19.915	−1.04%	7.586	−3.15%	4.355	0.11%
z = 2	21.315	−8.14%	7.524	−2.30%	4.353	0.17%
z = 3	19.798	−0.45%	7.468	−1.53%	4.350	0.22%
z = 4	20.265	−2.81%	7.436	−1.11%	4.350	0.22%
z = 5	19.011	**3.55%**	7.293	0.83%	4.337	**0.53%**
z = 6	21.127	−7.19%	7.424	−0.94%	4.350	0.23%
z = 7	20.153	−2.25%	7.418	−0.87%	4.346	0.32%
z = 8	20.785	−5.45%	7.304	**0.70%**	4.347	0.30%
z = 9	19.985	−1.39%	7.598	−3.31%	4.356	0.09%
z = 10	21.960	−11.41%	7.658	−4.12%	4.362	−0.05%

**Table 3:** Comparison of the average complexity performance of D-SCLF2 using $f_{\beta =0.4}$ and D-SCLF2 using $f_{\beta =0.4}^{line}$, with L=4, T=50, and different *z* values, for PC(512,256+24).

T = 50						
	1.5 dB		2.0 dB		2.5 dB	
	Lav	Rc	Lav	Rc	Lav	Rc
no z	44.065	0.00%	10.705	0.00%	4.609	0.00%
z = 1	46.856	−6.33%	11.527	−7.68%	4.534	1.62%
z = 2	46.932	−6.50%	11.299	−5.55%	4.577	0.70%
z = 3	44.038	0.06%	11.140	−4.06%	4.530	1.72%
z = 4	46.148	−4.72%	10.883	−1.66%	4.583	0.56%
z = 5	43.884	**0.41%**	11.119	−3.86%	4.533	1.65%
z = 6	47.267	−7.27%	10.352	**3.30%**	4.529	1.74%
z = 7	48.008	−8.95%	10.999	−2.74%	4.526	**1.80%**
z = 8	45.617	−3.52%	11.323	−5.77%	4.537	1.56%
z = 9	46.674	−5.92%	11.838	−10.59%	4.535	1.61%
z = 10	46.361	−5.21%	12.139	−13.39%	4.589	0.43%

**Table 4:** Comparison of the average complexity performance of the D-SCLF2 and the ALF2, for PC(512,128+24).

T = 15				T = 50			
Eb/N0 (dB)	D-SCLF2	ALF2	Rc	Eb/N0 (dB)	D-SCLF2	ALF2	Rc
1.25	11.379	11.093	2.51%	1.25	21.091	21.879	−3.74%
1.5	7.771	6.693	13.87%	1.5	11.505	10.256	10.86%
1.75	5.622	3.820	32.04%	1.75	6.878	5.093	25.95%
2	4.645	2.368	49.01%	2	5.062	2.789	44.92%
2.25	4.233	1.640	61.25%	2.25	4.354	1.749	59.83%
2.5	4.083	1.292	68.35%				

**Table 5:** Comparison of the average complexity performance of the D-SCLF2 and the ALF2, for PC(512,256+24).

T = 15				T = 50			
Eb/N0 (dB)	D-SCLF2	ALF2	Rc	Eb/N0 (dB)	D-SCLF2	ALF2	Rc
1.75	12.015	11.872	1.19%	1.75	21.701	22.179	−2.20%
2	7.355	6.109	16.94%	2	10.705	9.476	11.48%
2.25	5.192	3.171	38.93%	2.25	6.116	4.072	33.43%
2.5	4.360	1.878	56.94%	2.5	4.609	2.056	55.40%
2.75	4.099	1.335	67.43%				

**Table 6:** Comparison of the average complexity performance of the D-SCLF2 and the ALF2, for PC(512,384+24).

T = 15				T = 50			
Eb/N0 (dB)	D-SCLF2	ALF2	Rc	Eb/N0 (dB)	D-SCLF2	ALF2	Rc
3	9.518	9.211	3.23%	3	16.572	17.617	−6.30%
3.25	6.071	4.209	30.67%	3.25	8.174	6.411	21.57%
3.5	4.570	2.150	52.94%	3.5	4.954	2.571	48.10%
3.75	4.141	1.404	66.09%	3.75	4.195	1.478	64.77%

## Data Availability

Not applicable.
